# Compensation for coarticulation despite a midway speaker change: Reassessing effects and implications

**DOI:** 10.1371/journal.pone.0291992

**Published:** 2024-01-12

**Authors:** Navin Viswanathan, Ana Rinzler, Damian G. Kelty-Stephen

**Affiliations:** 1 Department of Communication Sciences & Disorders, The Pennsylvania State University, State College, Pennsylvania, United States of America; 2 Haskins Laboratories, New Haven, Connecticut, United States of America; 3 Department of Psychology, Rutgers University, New Brunswick, New Jersey, United States of America; 4 Department of Psychology, State University of New York-New Paltz, New Paltz, New York, United States of America; Università del Salento: Universita del Salento, ITALY

## Abstract

Accounts of speech perception disagree on how listeners demonstrate perceptual constancy despite considerable variation in the speech signal due to speakers’ coarticulation. According to the spectral contrast account, listeners’ compensation for coarticulation (CfC) results from listeners perceiving the target-segment frequencies differently depending on the contrastive effects exerted by the preceding sound’s frequencies. In this study, we reexamine a notable finding that listeners apparently demonstrate perceptual adjustments to coarticulation even when the identity of the speaker (i.e., the “source”) changes midway between speech segments. We evaluated these apparent across-talker CfC effects on the rationale that such adjustments to coarticulation would likely be maladaptive for perceiving speech in multi-talker settings. In addition, we evaluated whether such cross-talker adaptations, if detected, were modulated by prior experience. We did so by manipulating the exposure phase of three groups of listeners by (a) merely exposing them to our stimuli (b) explicitly alerting them to talker change or (c) implicitly alerting them to this change. All groups then completed identical test blocks in which we assessed their CfC patterns in within- and across-talker conditions. Our results uniformly demonstrated that, while all three groups showed robust CfC shifts in the within-talker conditions, no such shifts were detected in the across-talker condition. Our results call into question a speaker-neutral explanation for CfC. Broadly, this demonstrates the need to carefully examine the perceptual demands placed on listeners in constrained experimental tasks and to evaluate whether the accounts that derive from such settings scale up to the demands of real-world listening.

## Introduction

The question of how listeners demonstrate stable perception despite an acoustic signal that varies considerably due to factors such as speaking rate, talker identity, the listening environment etc. is a fundamental, yet unanswered, question in speech perception. One source of variability is due to coarticulation in speech production, a process which alters the acoustic characteristics of a given speech segment depending on its neighboring segments. This source of variability is inescapable because speech is always coarticulated, and this variability is present even when other factors (speaker identity, environment, speaking rate etc.) are held constant. Consequently, successful speech perception requires that listeners adjust their perceptual patterns according to coarticulatory context.

An example of such *compensation for coarticulation* (CfC) in which listeners categorizing members of a [ga]–[da] continuum report more [ga] responses after the precursor [al] than after [aɹ]. Mann [1] interpreted these perceptual shifts as evidence of listeners’ attunement to speech gestures. Specifically, by this gestural explanation, listeners compensate for the acoustic variation resulting from the change in the second syllable’s place of constriction due to coarticulation with the first. Alternatively, according to the General Auditory framework [2], coarticulation of segments in speech production leads to spectral assimilation (i.e. reduced frequency differences) across segmental boundaries [3]. However, speech perceivers overcome this assimilation by experiencing *spectral contrast* that accentuates differences at a strictly auditory level. For instance, target segment [ga] and precursor [aɹ] have relatively low F3 frequencies compared to [da] and [al]. Hence, the *high* F3 in [al] leads listeners to be more likely to hear the following F3 as *lower* and hence more [ga]-like. The opposite pattern is expected when they hear [aɹ] with relatively lower F3. By this account, CfC results from auditory consequences rather than from perceiving coarticulatory effects of gestures in speech production (cf. [1]). It is important to note that spectral contrast has also been hypothesized to underlie listeners’ ability to adjust to acoustic changes accompanying variability due to multiple takers, i.e., *talker normalization* [4–6]. Per this account, listeners perceiving longer segments of speech (e.g., sentences) track the long-term average of critical frequencies and demonstrate category boundary shifts on target segments predicted by the contrastive effects expected from long term spectral averages (global spectral contrast). In the current set of experiments, we focused on evaluating the spectral contrast account of CfC that relies on contrast effects exerted by neighboring segments (local spectral contrast). However, in the discussion, we will consider the implications of the current findings and other recent ones on both sorts of spectral contrast accounts.

Lotto and Kluender [3] offered two lines of support for a spectral contrast explanation of CfC. First, they demonstrated that listeners shifted perception of target speech even whenpure nonspeech tone precursors, devoid of gestural information but retaining assumed critical frequencies, were used. Tone-analogue effects extend across phonetic contexts and in different listener populations such as native listeners, non-native listeners, listeners who are hard of hearing etc. However, a series of studies have systematically called into question whether such locally- contrastive tonal effects accurately capture processes responsible for CfC. In particular, these studies have demonstrated CfC effects both in the direction predicted by spectral contrast (e.g., [7]) as well as against the direction predicted by spectral contrast (e.g., [8]). Other studies have found CfC effects in the absence of spectral contrast (e.g., [9, 10]), failed to find CfC effects despite the presence of spectral contrast [7, 11] and demonstrated different time course of effects for speech and non-speech contexts matched in critical frequencies [12]. Taken together, these findings dispute the relevance of local spectral contrasts in predicting listeners’ CfC behavior.

In the current study, we focus on the second line of support for a spectral contrast explanation for CfC suggested by Lotto & Kluender [3]. They demonstrated that listeners shifted perception of the [da]-[ga] target continuum, as long as the critical contrastive relations were maintained, *whether or not* the same speaker produced precursor syllables [al] or [aɹ]. They reasoned that such source-neutral effects of spectral contrast offer evidence against the explanation that CfC results from the perception of vocal gestures. They argued that if that were the case, listeners would not compensate for coarticulation across speakers. This line of reasoning has been used in subsequent studies of both local [13, 14] as well as global spectral contrast effects [4, 15]. Furthermore, apparent source-neutral effects on speech perception extend beyond compensation for coarticulation, inviting the similar interpretation that speech perception systems attend to signal regularities irrespective of (or prior to) speech-signal source [16, 17].

However, such hypothesized source-neutral effects require further scrutiny. Our ability to navigate typical listening situations that often involve multiple simultaneous speakers (termed the cocktail party problem [18]) suggests a fundamental capacity to attune to a conversation partner among other speakers in the same room. It is unclear how speech perceivers could demonstrate perceptual stability in multi-talker settings if the underlying mechanisms proposed to overcome such variability operate indiscriminately across speakers in the room. Such source-neutral processes operating on acoustics alone (such as F3 or duration) appear to be ill-suited to overcoming the challenges that such multi-speaker settings would pose. Indeed, if true, they may lead listeners to compensate for the wrong speaker’s regularities and contradict the known abilities of perceptual stability that human listeners demonstrate in such settings.

Given this rationale and the claims of the spectral contrast account, it is vital to reexamine CfC in the context of talker change. Studying CfC in this context presses an incisive question to the heart of long-standing debates about how listeners compensate for coarticulation in more typical listening conditions. If talker change does not affect CfC, crucially, it means listeners may still rely on general-auditory aspects of the speech signal in phonetic processing despite idiolectical acoustic variation that is often present in multi-talker settings. On the other hand, if talker change weakens CfC, then, from the gestural account, it would indicate that listeners’ ability to draw stable perception of variable speech relies on attuning to the invariants in acoustic signal that result from the causal vocal tract gestures.

One possible explanation for such across-speaker CfC effects could be that these effects are artifactual consequences of testing listeners under controlled but contrived experimental settings. For instance, under such settings, Vitevitch [19] demonstrated *change deafness effects*. In a pair of experiments, he found that greater than 40% of listeners failed to even detect a talker change in the middle of an experimental session. More importantly, the listener’s detection of a change in talker affected how the listener processed the stimuli after the talker changed. Despite the presence of CfC in the across-talker condition, Lotto and Kluender [3] did not examine whether the size of these effects may have been smaller than in the within-talker condition. Minimally, we suggest that cross-talker CfC effects warrant a more thorough examination before we accept them as indicative of the fundamental speech processes/architecture. Similar patterns of CfC under talker change must not be conflated with equal strength of CfC, and revisiting these effects may offer critical clues regarding how listeners cope with coarticulation in settings involving different talkers.

In the current study, we elaborate Lotto and Kluender’s [3] test by using three different exposure conditions between groups. Each group was assigned a task that required focus on the precursor, or on the target, or on the detection of talker change itself. The rationale of this current manipulation is to assess whether we replicate the critical finding of cross-talker CfC and then examine whether such effects endure when the listeners’ experience with the stimuli is altered. To clarify, each condition uses the same test stimuli in the test block but varies the experience that listeners have had with these stimuli in an earlier exposure block. We then test within- and across-speaker CfC in these three conditions both to evaluate the robustness of mixed-talker CfC effects across these conditions as well as to evaluate whether the focus of the task affects the resulting pattern of CfC (see Table 1 for summary of conditions). If listeners’ CfC patterns depend solely on spectral relations between the precursor and target, then all groups should show perceptual shifts irrespective of whether the speaker shifted in mid-sequence of the disyllable [3]. In fact, talker change should only increase spectral contrast: the spectral contrast between precursor and target syllables is greater for the female precursor leading to the prediction of *larger* CfC shifts in across-talker (female-male) than within-talker (male-male) condition (Table 2; [20]; cf. [9]). However, if purely spectral relations do not underlie CfC and listeners do not disregard source information, the across-talker compared to the within-talker condition should produce *smaller or no* CfC effects. Finally, while both accounts posit that the same mechanisms (attunement to gestural dynamics or spectral contrast) to cope with variability due to coarticulation and talker differences, neither suggests that one form of variability (e.g., due to talker) is handled before the other (e.g., coarticulation).

**Table 1:** Structure of experimental conditions.

Between-Subjects’ Condition	Exposure Block Stimuli	Exposure Block Task	Test Block Stimuli (common to all conditions)	Test Block Task (common to all conditions)
Baseline	Same as the Test Block	Classify if the first syllable is an “al” or “ar”	Standard disyllables. Precursors consisted of naturally produced [al] and [aɹ] productions by male and female speakers. Standard eleven-step [ga]-[da] resynthesized continuum produced by a male speaker	Classify if the target (second) syllable is a “ga” or a “da”
Explicit Talker-Change	Same as the Test Block	Classify if the speaker changed midway during each trial		
Implicit Talker-Change	Same as the Test Block; inter-syllable gap was varied such that the precursor and target partially overlapped in some trials	Classify if the first syllable is an “al” or “ar”		

**Table 2:** Offset frequencies of precursor syllables.

Talker Sex	Syllable	Mean F0 (Hz)	F2 offset (Hz)	F3 offset Hz	F4 offset Hz
Female	[al]	210	1200	3800	4000
Female	[aɹ]	210	2000	2300	3400
Male	[al]	120	1060	2600	3600
Male	[aɹ]	120	1350	1800	3050

## Materials and methods

### Subjects

Eighty-one native English-speaking participants with normal hearing from the University of Kansas provided informed consent through an oral consent procedure according to the Institutional Review Board at the University of Kansas and received course credit for their participation. This study was approved under protocol HSCL#3059. This sample size, assuming a moderate effect size at an alpha level of .05 per past studies [3], provided a power of 90%.

### Materials

By manipulating F3-onset frequency for syllables [ga] (1800Hz) to [da] (2800 Hz), we created an 11-step continuum of CV syllables ranging perceptually from [ga] to [da]. Each CV continuum member had 2500-Hz steady-state value and a 110-Hz F0 average. The first, second and fourth formants were identical for each step: F1 increasing from 500Hz to 800Hz, F2 decreasing from 1600Hz to 1200Hz, and F4 steady at 3300Hz. Each syllable was 215ms. The formant frequencies, used for resynthesis, were modeled after a canonical male talker. Precursors consisted of natural male and female productions of [al] and [aɹ]. These acoustic characteristics modeled the precursors used in Experiment 1 of [3]. The table of frequency offsets for each precursor syllable for each speaker is provided in Table 2. Note that in past studies, we have replicated the typical CfC shifts using these stimuli (within-talker CfC effect, average shift of 7.62%, p < .001, [7]; and the across-talker CfC effect, average shift of 2.5%, p < .05 [21]). Speech tokens consisted of VC precursors and CV syllables with constant 80ms silence between precursor and syllable. The standard tokens were test items for all groups in the testing block. As a result, participants heard 44 disyllables (2 talkers × 2 precursor × 11 targets) with a total duration of 590ms per disyllable. The same stimuli appeared in the exposure block for both baseline and explicit speaker-change groups. In the exposure block for the implicit speaker-change group, the gap between precursors ranged from -50% of precursor duration (approx. 200 ms overlap) to +50% (approx. 200 ms gap between syllables). Overlapping disyllables only appeared in the mixed-sex pair to highlight talker change implicitly. The mixed design manipulated talker change (mixed-sex vs. single-sex), precursor ([al] vs. [aɹ]) and continuum steps within subjects and manipulated preceding task (baseline vs. explicit talker-change, vs. implicit talker-change) across subjects.

### Procedure

Subjects were randomly assigned to one of three groups (again see Table 1). Within each group, listeners completed exposure, categorization of the target endpoints, and a test block. Critically, while the exposure block was different across the three groups, the latter two blocks were identical. In the exposure block, the baseline group heard the disyllable stimuli and categorized whether the first syllable was an [al] or [aɹ]. The explicit talker-change group indicated whether there was a talker change midway through the disyllable. The implicit talker-change group also performed an [al] or [aɹ] categorization with a varying gap as noted above. After the exposure block, all three groups completed endpoint target identification to familiarize themselves with the target syllables. Finally, all groups completed an identical CfC test block. Subjects heard steps of the target continuum tokens preceded by either [al] or [aɹ] produced by either a female or a male speaker. They reported whether the second syllable they heard was a [ga] or [da].

### Data analysis

We modeled the responses in the CfC test block using mixed effects logistic regression, using a generalized linear model with a binomial linking function using glmer() in R [22] software library “lme4” [23]. All models included centered trial-varying predictors, that were contrast coded [24], Precursor (equaling -0.5 for [al] and 0.5 for [aɹ]), continuum Step (values ranging from -5 to +5), Talker Consistency (equaling -0.5 when talker changed and + 0.5 when it remained the same), Condition (equaling 1, 2, or 3 for trials completed in *baseline*, *explicit*, or *implicit* conditions, respectively). We used categorical coding for Condition by using as.factor() within glmer syntax and respected Bolker’s convention of model convergence when maximum absolute gradient was < 0.001 [25]. The data files and the analyses scripts are available from this link: https://osf.io/b7xjv/?view_only=545e310fa43749a59bcb905e859e5d9c

## Results

**Fig 1:**
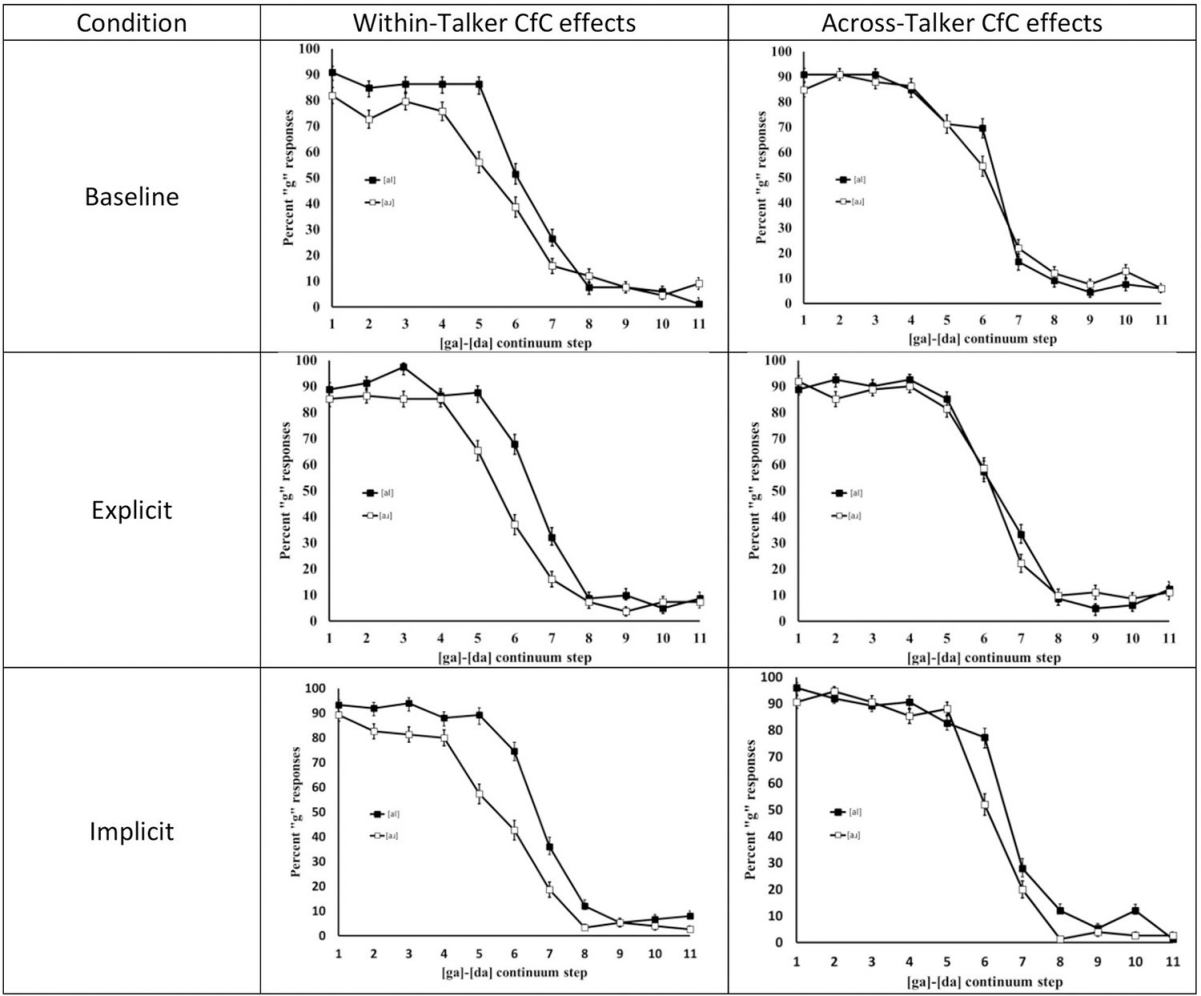
Compensation for coarticulatory patterns across all conditions.

Excluding five participants who failed to respond to more than 10% of trials left 24 subjects in the baseline and 26 subjects each in explicit and implicit speaker-change groups. Fig 1 depicts listeners’ identification responses as a function of continuum step and precursor for each condition. A visual inspection of the results reveals a clear and consistent pattern across all three conditions: listeners demonstrate a shift in target identification as a function of the precursor only when there is no talker change (the left panels). When the talker changed from a female speaker to a male midway through the utterance, unlike Lotto & Kluender [3], the precursors fail to produce clear shifts in target identification (the right panels). Critically, this demonstrates that irrespective of particulars of the exposure block that differed between the groups, there were no cross-talker CfC effects in the test block that was identical for the three groups.

Percentage [ga] responses are plotted on the y axis. Lines with solid markers indicate responses after [al] and the lines with unfilled markers indicating responses after [aɹ]). The panels on the left indicate clear boundary shifts (indicated by the separation of lines) in the within-talker conditions. These effects diminish substantially in the right panels that depict performance in the across-talker conditions. Error bars denote 95% confidence intervals. These data were submitted a logistic mixed effects model with a binomial distribution specified. Precursor, Step, Talker consistency, and Precursor X Talker consistency were entered as fixed factors. By-subject slopes for step and talker consistency and by-subject intercepts were included as random effects. This structure was the maximal model that still allowed for model convergence. Please refer to supplementary materials for a model that included group as a fixed factor and only random intercepts that show qualitatively similar effects for all critical variables. Results of binomial modeling of the likelihood of “GA” responses for any given trial confirm the observed patterns (Table 3).

**Table 3:** Omnibus logistic mixed regression model across all groups of listeners.

Predictor	*B*	*Standard Error*	z	p
Intercept	- 0.052	0.070	-0.743	0.46
Prec	-0.527	0.060	-8.725	< 0.0001
Step	- 0.920	0.055	-16.565	< 0.0001
Talker Consistency	-0.372	0.105	-3.549	0.0003
Prec X Talker Consistency	-0.682	0.120	-5.676	< 0.0001

Our analyses confirms the expected effect that varying step of the continuum from [ga] to [da] resulted in reliably decreased the logarithmic probability of participants reporting a “GA” (*B* = -.523, *SE* = .012, *z* = -16.56, *p* < .0001). Next we also replicated the classic CfC effect that the logarithmic probability of a listener reporting ga went down when the precursor changed from [al] to [aɹ] (*B = -*.*053*, *SE =* .*06*, *z = -8*.*725*, *p <* .*0001*). Finally, critically for the current study, there was clear interaction between Precursor and Talker consistency (*B = -*.*682*, *SE =* .*12*, *z = -5*.*676*, *p <* .*0001)*. This confirms the visually apparent pattern of smaller effects of the precursor when the talkers were mismatched.

We explored this interaction using two sets of follow up analyses. First, we analyzed each group separately (Table 4). All effects, including the critical Prec X Talker Consistency effect held for each of the three groups indicating that in all three conditions, the CfC effects were weaker when the talkers were mismatched.

**Table 4:** Follow up analysis of critical effects by group.

**Exposure Group: Predictor**	***B***	***Standard Error***	**z**	**p**
Intercept	- 0.225	0.092	-2.453	0.014
Prec	-0.336	0.102	-3.303	0.001
Step	- 0.645	0.0225	-28.708	< 0.0001
Talker Consistency	-0.301	0.193	-1.563	0.12
Prec X Talker Consistency	-0.605	0.204	-2.973	0.002
**Explicit Group: Predictor**	***B***	***Standard Error***	**z**	**p**
Intercept	0.003	0.100	0.031	0.98
Prec	-0.404	0.096	-4.222	< 0.0001
Step	-0.696	0.022	-31.722	< 0.0001
Talker Consistency	-0.225	0.112	-2.008	0.045
Prec X Talker Consistency	-0.617	0.191	-3.228	0.001
**Implicit Group: Predictor**	***B***	***Standard Error***	**z**	**p**
Intercept	-0.069	0.137	-0.501	0.62
Prec	-0.748	0.109	-6.881	< 0.0001
Step	-0.825	0.027	-29.997	< 0.0001
Talker Consistency	-0.249	0.207	-1.204	0.23
Prec X Talker Consistency	-0.662	0.214	-3.094	0.002

Finally, we assessed whether the CfC effects endured when we examined only those trials in which there was a midway talker change (Table 5). Critically, the effect of precursor was no longer significant (p = .77) suggesting that overall, there were no CfC effects when the target changed. To further explore the apparent separation of curves in group 3 as seen in Fig 1, we examined the interaction of the PrecXGroup3. This was significant (p = .047) indicating that the precursor effect for that group was significantly different from the exposure group. Nevertheless, our previous analyses (Table 4) confirm that this precursor effect is significantly weaker (p = .002) than when the talker remained the same for this group.

**Table 5:** Follow up analyses of only trials in which there was a midway talker change.

Talker Change: Predictor	*B*	*Standard Error*	z	p
Intercept	0.103	0.154	0.66	0.51
Prec	-0.045	0.153	-0.293	0.769
Step	-1.060	0.077	-13.80	< 0.0001
Group 2	0.171	0.199	0.861	0.380
Group 3	0.067	0.209	0.321	0.748
PrecXGroup2	-0.062	0.208	0.300	0.764
PrecXGroup 3	-0.438	0.221	-1.982	.048

## Discussion

We reexamined the finding that listeners demonstrate perceptual boundary shifts even when different speakers produce the precursor and the target [3]. This finding has been used as evidence for a spectral-contrast explanation of CfC and, more generally, for a source-neutral account of how listeners overcome speech variability. We reevaluated this finding in order to determine whether the altering listener’s prior experience with these unnatural stimuli would change their CfC patterns. We tested three groups of listeners, one explicitly attending to the speaker-change (explicit speaker-change group), another hearing precursor and the target overlapping in a subset of mixed-sex trials (implicit speaker change group), and a third having extended exposure to these stimuli (baseline group). Consistently, and remarkably, our results indicated that in each of the three groups, irrespective of exposure, we detected clear CfC effects only when the speaker did not change across the disyllables. Taken together, these findings suggest that listeners demonstrate context-appropriate CfC shifts only when there is no speaker change. Importantly, these results suggest that listeners exposed to the mixed-talker disyllables do not disregard the source of regularities in speech and do not operate on contrast alone.

More generally, the current results demonstrate that findings such as ones indicating source-neutral speech processing requires reexamination in terms of the experimental demands placed on the listener and whether they generalize to conditions of typical listening. Even in the original study, as noted, the mixed talker effects appear to be smaller indicating that listeners do not appear to disregard speaker information entirely. As noted, this effect is predicted to be larger from a spectral contrast perspective based on the offsets reported in Table 2. In our current study, we suspect that we did not find cross-talker CfC effects because our listeners were able to attune to the speakers due to increased exposure to the disyllables in all three conditions. One possibility is that the current set of studies indicate that any talker normalization processes (or more generally attunement to talker variation) could precede coarticulatory compensation mechanisms. A similar suggestion has been made for rate normalization processes [17]. Further research is required to explore this possibility and its implications for the accounts in question. For now, we conclude that a completely source-neutral mechanism (that ignores vocal tract properties by the gestural account) is unlikely to be responsible for CfC.

Further, while these studies use the classic paradigm to evaluate this questions, recent studies using more sophisticated paradigms appear to offer convergent support. For instance, the study of spectral contrast effects in a related domain of talker variability may help to frame our current results and inform future directions of research into CfC. Specifically, the spectral contrast hypothesis is that listeners track global spectral averages in context speech which is then used by the auditory system to contrastively enhance the perception of subsequent target speech. Such contrastive effects enable listeners to adjust for the effects of vocal tract size differences on the resulting acoustics. Per this account, this negates the need for listeners to attune to the gestural dynamics or vocal tract size differences directly. A recent study in this domain that examines such global spectral contrast effects in multi-talker settings is especially relevant to our current study [15]. While their focus was on trying to understand the attentional effects on observed contrast effects, we focus on two findings that are critical to our discussion. First, using single talker streams, they demonstrated that the size of contexts effects on target perception (spectral contrast effects by their account) was significantly reduced when there is a midway talker change compared to the same talker condition (Experiment 1B vs. 1A in [15]). Second, when multiple talkers were simultaneously presented, a midway talker change completely eliminated any spectral contrast effects. These findings lead the authors to this key conclusion “Thus, we show that the contribution of spectral contrast effects to more naturalistic listening conditions may be modest, highlighting the need for studying the processes involved in speech perception in their natural habitat” [15] (pp. 1331).

Despite approaching the problem of speech variability from a different theoretical perspective, focusing on a different phenomenon, and utilizing a different paradigm, we arrive at a similar conclusion from our current studies. Any explanations regarding fundamental aspects of speech perception architecture derived from artificial experimental settings must be subject to an ecological smell test. Hypothesized mechanisms from such studies must serve the perceivers in everyday listening. Given that listeners typically confront considerable signal variability amidst multiple competing sources, explanations such as spectral contrast that operate solely on surface level acoustic characteristics may have difficulty generalizing to ecologically-typical settings such as ones with multiple competing talkers. Such settings not only illustrate the robustness of human speech perception but also serve as useful testing grounds for speech perception accounts.
